# Sex differences in clinical trials of ALRV5XR treatment of androgenetic alopecia and telogen effluvium

**DOI:** 10.3389/fmed.2022.918058

**Published:** 2022-08-04

**Authors:** Peter R. Feldman, Oriel J. Feldman, Jaime Guevara-Aguirre, Klaus M. Fiebig

**Affiliations:** ^1^Arbor Life Labs, Toronto, ON, Canada; ^2^Faculty of Science, Wilfrid Laurier University, Waterloo, ON, Canada; ^3^College of Medicine, Universidad San Francisco de Quito, Quito, Ecuador; ^4^Faculty of Health, Medicine and Life Sciences, Maastricht University, Maastricht, Netherlands; ^5^Institute of Endocrinology, Metabolism, and Reproduction, Quito, Ecuador; ^6^College of Medicine, University of Florida, Gainesville, FL, United States

**Keywords:** ALRV5XR, androgenetic alopecia, pattern hair loss, hair restoration, telogen effluvium, regenerative medicine, miniaturization reversal, hair regrowth dynamics

## Abstract

**Introduction:**

ALRV5XR treatment of androgenetic alopecia (AGA) and telogen effluvium (TE) has early evidence of regenerating a normal scalp hair phenotype in both sexes.

**Design:**

We performed two 24-week double-blinded placebo-controlled comparison trials, one in each sex, on the ALRV5XR treatment effect on hair regeneration, in AGA and TE, in 92 AGA subjects (24 also had TE). Forty-six women (age 24–64 years) and 46 men (age 22–63 years) were randomized 1:1 to either ALRV5XR or placebo regimens (one b.i.d. oral capsule and daily administration of shampoo, conditioner, and follicle serum).

**Evaluation:**

*Primary outcomes*: Absolute and relative changes in terminal hair (TH) density. *Secondary outcomes*: Response rate, changes in vellus hair (VH) density, TH/VH ratio, hair diameter, growth, and shedding rate.

**Results:**

Forty-one women (20 ALRV5XR, 21 placebo) and 36 men (17 ALRV5XR, 19 placebo) completed the trials. TH outcome was evaluable for 18 and 21 women and 11 and 11 men (ALRV5XR, placebo, respectively). Efficacy in women: 30.1 THs/cm^2^ (*p* = 0.0002) and 19.7% (*p* = 0.0016). Efficacy in men: 21.0 THs/cm^2^ (*p* = 0.0014) and 16.4% (*p* = 0.0012). 66.7% of women and 100% of men responded to ALRV5XR. TH/VH ratio for men increased 33.0% (*p* = 0.0033). Growth rate in women increased by 30.7 μm/24 h (*p* < 0.0001) and 10.0% (*p* < 0.0001). There were no adverse events reported.

**Conclusion and relevance:**

ALRV5XR induced significant regrowth of TH. Accelerating regrowth by reactivation of dormant telogen follicles were the dominant effects in women. Thickening of miniaturized hair and regrowth of dormant telogen follicles contributed equally to the increased TH seen in men (see Graphical Abstract).

## Graphical Abstract

Sex differences in hair regrowth with ALRV5XR. Blue ovals are absolute Terminal or Vellus hair counts at baseline. Green ovals represent increases of hair density, and red ovals are reductions. (a) Changes in dormant follicles account for less empty follicles due to new hair regrowth. (b) Vellus Hairs include True Vellus, Vellus-Like and New Hairs. Arrows in black show new hairs from dormant follicles regrowing into Vellus or Terminal hairs, and Vellus hairs thickening into Terminal hairs. Arrows in red are reductions in Vellus hairs or dormant follicles. Statistical significance between ALRV5XR and placebo: *P < 0.05; **P < 0.01; ***P < 0.001. 
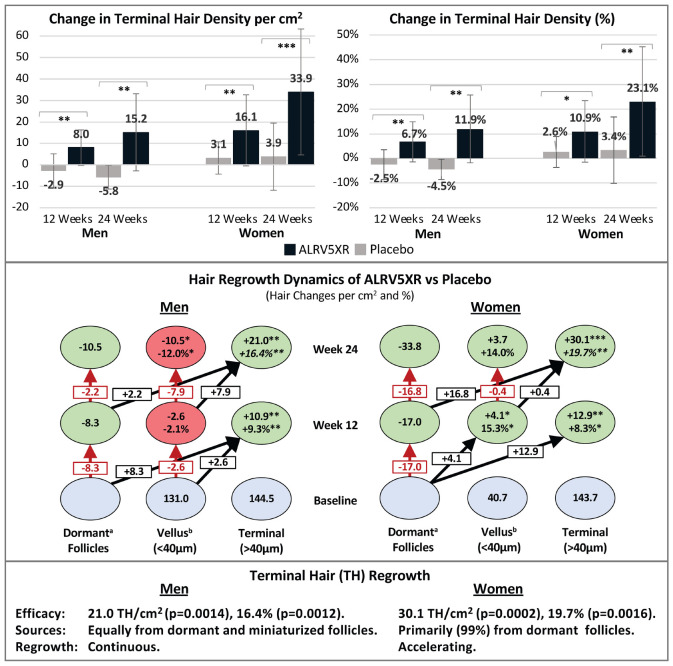

## Introduction

ALRV5XR is a novel natural treatment of standardized botanicals, vitamins, and minerals designed to regenerate a normal hair phenotype by targeting more than 20 molecular pathways in the hair follicle (HF) linked to HF stem cell activation, promotion of healthy hair growth, and normal HF physiology. It was designed to modulate multiple cytokines, growth factors, and hormone mediators while relieving stress on the HF and, more specifically, to upregulate growth promotion pathways of the HF while inhibiting targets linked to hair loss. Among these actions, the Wnt/β-Catenin cascade in HF stem cells and dermal papilla cells is regarded as the dominant effector for new hair formation and maintenance of growth. Similarly, ALRV5XR includes botanicals known to modulate growth factors such as FGF-7, FGF-18, HGF, IGF, KGF, and VEGF, which are well-characterized in the induction and maintenance of anagen. Additionally, DHT and androgen regulator 5α -Reductase, as well as cytokines such as mTORC1, PKC, PPARγ, Shh, BMP-4, COX, INF-α, INF-γ, Interleukins 1b, 6, and 12, NF-κβ, Prostaglandin D2, TNF-α, and TGF-β, are all targets which have been reported to have been influenced directly or indirectly by one or more ingredients of ALRV5XR. ALRV5XR was developed for use as oral supplementation and topical applications in shampoo, conditioner, and serum (see Supplementary Material).

We previously reported clinically significant terminal hair (TH) regrowth in treatment with ALRV5XR with no adverse signals or events in two double-blinded randomized placebo-controlled trials (RCTs) in women and men with androgenetic alopecia (AGA) and telogen effluvium (TE) (1, 2). We report herein the pertinent similarities and differences in outcomes of the trials between women and men. We also report new secondary trichometric data that provides insight into the different hair regrowth dynamics observed in women and men. In these RCTs, TH was reported as hair with a diameter > 40 μm, and vellus or vellus-like hair (VH) with a diameter < 40 μm (1, 2).

We hypothesize that ALRV5XR, a highly effective treatment in regenerating and maintaining TH in women and men, achieves these effects by both reactivating dormant telogen HFs and thickening of AGA miniaturized (vellus-like) hairs.

## Materials and methods

Two RCTs were conducted in women and men with AGA over 24 weeks. One hundred fifty-one volunteer subjects between the ages of 18 and 65 were screened for eligibility resulting in 92 healthy subjects being enrolled. All subjects were of Fitzpatrick skin type I-VI and had AGA. A primary or secondary diagnosis of AGA or TE was assigned. Forty-six women having Ludwig classification I-1–II-2 and 46 men with Hamilton-Norwood classification I-VII were randomized 1:1 to ALRV5XR or placebo treatment groups. Scalp and physical evaluation, vertex phototrichograms, blood and urine samples were taken at baseline, 12 and 24 weeks. Subjects were given a masked treatment of ALRV5XR or placebo as one oral capsule (842 mg) taken twice per day and daily administration of shampoo (3–7 ml), conditioner (3–7 ml), and follicle serum (1 ml) over the 24-week study duration (see Supplementary Material). Primary outcomes were absolute and relative changes in TH densities. Secondary outcomes were response rates, changes in VH density, TH/VH ratios, hair diameter, and growth and shedding rates. The trials were conducted in a community clinic in San Francisco, United States, between April 3 and October 23 of 2018. Primary findings were published with CONSORT adherence in July (women) and October (men) 2021. Trial registration with ClinicalTrials.gov was NCT04450589 and NCT04450602.

## Results at 24 weeks

### Participants and baseline trichometry

Ninety-two healthy subjects were enrolled in the two RCTs, with 46 women and 46 men randomly assigned (1:1) into ALRV5XR or placebo groups with mean(±SD) age 50.1(±9.1) and 51.6(±10.7) years for women, and 48.3(±7.9) and 45.7(±12.9) for men, respectively. Forty-one subjects completed the women’s trial (20 ALRV5XR, 21 placebo), and 36 subjects completed the men’s trial (17 ALRV5XR, 19 placebo), all evaluable for safety. All subjects were diagnosed with AGA, and 23 women (8 ALRV5XR, 15 placebo) also had TE. Two women were non-compliant, resulting in 18 ALRV5XR and 21 placebo evaluable for TH outcomes, and 11 male subjects in each group were evaluable for TH outcomes. At baseline, both sexes had a similar TH density of approximately 140 THs/cm^2^, while men had 3-4 times more VH than women. TH/VH ratios were 3–4.5 for women and 1–1.1 for men. Total hair density for women at baseline was approximately 90 Hairs/cm^2^ less than the men (185 vs. 275 Hairs/cm^2^). Mean diameters of all hairs and THs for women were 61 and 69 μm, and for men, 43 and 54 μm, respectively. Hair growth rate for women was 307 μm/24 h, and for men, 232 μm/24 h (see Supplementary Table S1).

### Primary results and safety

**FIGURE 1:**
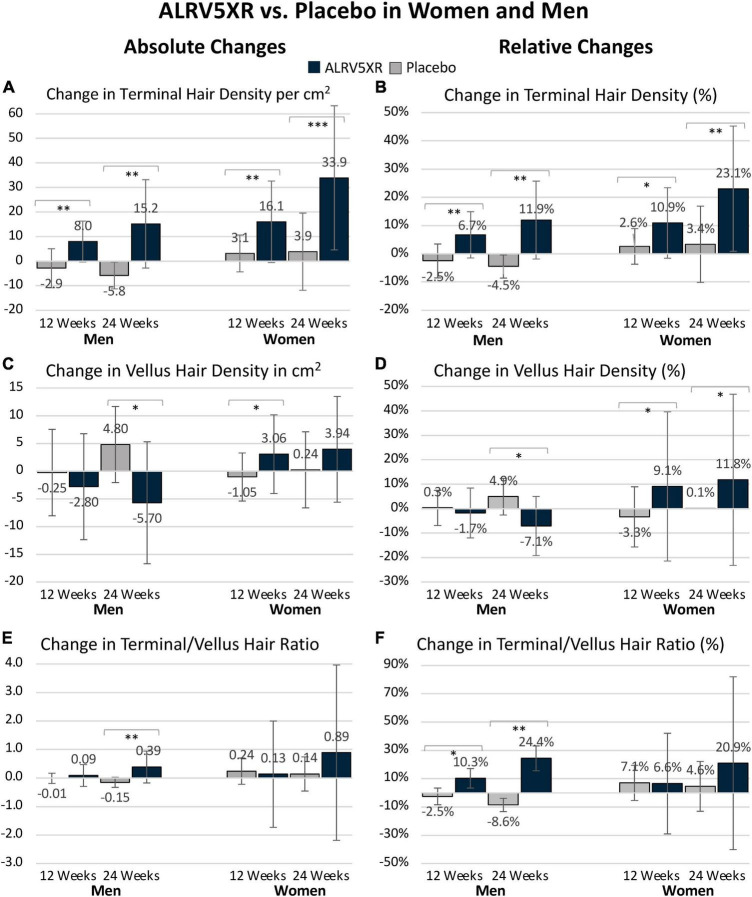
ALRV5XR vs. Placebo in women and men with AGA and TE over 24 weeks. **(A)** Absolute Changes (AC) in Terminal Hairs (TH) per cm^2^; **(B)** Relative Changes (RC) in TH in percent (%); **(C)** AC in Vellus Hair (VH); **(D)** RC in VH%; **(E)** AC in TV/VH ratio; **(F)** RC in TH/VH ratio in %. Statistical significance of ALRV5XR vs. Placebo: *P < 0.05, **P < 0.01, and ***P < 0.001. Error bars are 95% confidence intervals.

ALRV5XR increased TH density in both women and men with statistical significance compared to placebo. Women increased their average TH density by 30.1 THs/cm^2^ (*p* = 0.0002) and 19.7% (*p* = 0.0016) with odds ratio (OR) 2.7. Men increased their average TH density by 21.0 THs/cm^2^ (*p* = 0.0014) and 16.4% (*p* = 0.0012) with OR 87.4 (Table 1 and Figure 1). Having a similar TH density at baseline, the absolute increase in men’s TH was 2/3 of the women, and the percentage TH increase for men was 4/5 of women. Efficacy was clinically significant in subgroups of women with a primary diagnosis of AGA or TE, and each was similar to the efficacy of the overall cohort of women. The combined overall TH regrowth efficacy of the two RCTs was 26.3 THs/cm^2^ (*p* < 0.0001) and 18.2% (*p* < 0.0001). In both sexes, these results were not affected by age, ethnicity, hair loss pattern, Fitzpatrick skin type, or body mass index. No clinically relevant adverse signals or adverse events were reported (see Table 1 and Figure 1).

**TABLE 1:** Summary of results after 24 weeks.

Summary of efficacy results after 24 weeks of treatment between ALRV5XR and placebo								
	**Men**				**Women**			
		
**Efficacy**	**Δ-Mean**	**95% CI**	***P*-value**	**OR**	**Δ-Mean**	**95% CI**	***P*-value**	**OR**
		
**Hair density changes**								
Terminal (per cm^2^)^a^	21.0	9.2–32.8	0.0014	87.4	30.1	15.1–45.1	0.0002	2.7
Terminal (%)^a^	16.4	7.4–25.5	0.0012	87.4	19.7	8.0–31.4	0.0016	2.7
Terminal response rate (%)	100.0				67.0			
Vellus (per cm^2^)	−10.5	−19.1–1.9	0.0196	0.1	3.7	−1.6–9.0	>0.05	2.4
Vellus (%)	−12.0	−21.5–2.5	0.0158	0.1	11.7	−4.9–28.2	>0.05	2.4
Total (per cm^2^)	13.4	−0.9–27.7	>0.05	4.0	33.9	18.1–49.8	0.0001	4.2
Total (%)	5.0	−0.7–10.7	>0.05	4.0	18.6	8.8–28.4	0.0004	4.2
**Terminal/vellus ratio changes**								
Absolute	0.5	0.1–0.9	0.0097	81.0	0.7	−0.6–2⋅1	>0.05	0.8
Relative (%)	33.0	12.5–53.4	0.0033	81.0	16.3	−11.9–44.6	>0.05	0.8
**Hair diameter changes**								
Terminal (μm)	0.2	−2.9–3.3	>0.05	1.8	−0.7	−3.6–2.2	>0.05	1.8
Total (μm)	2.0	−0.6–4.7	>0.05	4.1	−1.2	−4.4–2.1	>0.05	1.2
**Growth rate changes**								
Absolute (μm per 24 h)	10.4	−0.3–17.6	0.0552	0.8	30.7	19.1–42.3	<0.0001	12.8
Relative (%)	4.8	−0.2–9.8	>0.05	0.8	10.0	6.1–14.0	<0.0001	12.8
**Shed rate changes**								
Absolute (Scalp per day)*^b^*	−127.2	−260.1–5.6	0.0594	0.2	22.9	−7.0–52.8	>0.05	0.3
Relative (%)	−21.5	−38.5—4.5	0.0158	0.2	21.8	−6.5–50.2	>0.05	0.3
		
**Safety**	***N***				***N***			
		
Adverse signals	0				0			
Adverse events	0				0			

^a^Primary objectives results.

^b^Average scalp area (700 cm^2^).

Δ-Mean, difference of means between ALRV5XR and placebo; OR, odds ratio; Vellus hair includes true vellus, vellus-like and new hair (all <40 μm in diameter); N, number of adverse signals or events.

### Secondary results

#### Response rates

Sixty seven percent of women responded to ALRV5XR compared to 100% of men. In general, the women responders regrew approximately double the amount of TH than the men. In the ALRV5XR women’s group, all responders increased TH density by 30 THs/cm^2^ or more, and 44.5% increased their TH density by at least 30%. In the ALRV5XR men’s group, 54.5% of responders increased TH by 10 THs/cm^2^ or more, and 18.2% increased TH by more than 25 THs/cm^2^. 45.5% of men increased their TH density by at least 10%, and 18.2% increased TH density by more than 20% (see Supplementary Table S2).

#### Regrowth rate

The TH regrowth rate in women accelerated during the second 12-week treatment period and was 133% of the first 12-week period. The men’s regrowth rate remained constant throughout the 24 weeks (see Table 1).

#### Terminal to vellus hair ratio changes

Vellus hair density increased without significance in women, while in men, VH density declined significantly by −10.5 VHs/cm^2^ (*p* = 0.0196) and −12.0% (*p* = 0.0158) with OR 0.1. Consequently, men’s TH/VH ratio increased by 33.0% (*p* = 0.0033) with OR 81.0. In women, the TH/VH ratio increased by 11.2% without significance (see Table 1 and Figure 1).

#### Total hair density regrowth

Women reported a significant increase in total hair density of 33.9 Hairs/cm^2^ (*p* = 0.0001) with OR 4.2 when comparing ALRV5XR to placebo, while men reported a non-significant increase in total hair density (see Table 1).

#### Growth and shedding rates

The growth rate for women treated with ALRV5XR increased by 30.7 μm/24 h (*p* = 0.0001) or 10.0% (*p* = 0.0001) when compared to placebo, and the men’s increase was borderline significant. Hair shedding rates in men decreased by 21.5% (*p* = 0.0158) and in women were trending down relative to the increased density but were not significant (see Table 1).

### Hair regrowth dynamics results

**FIGURE 2:**
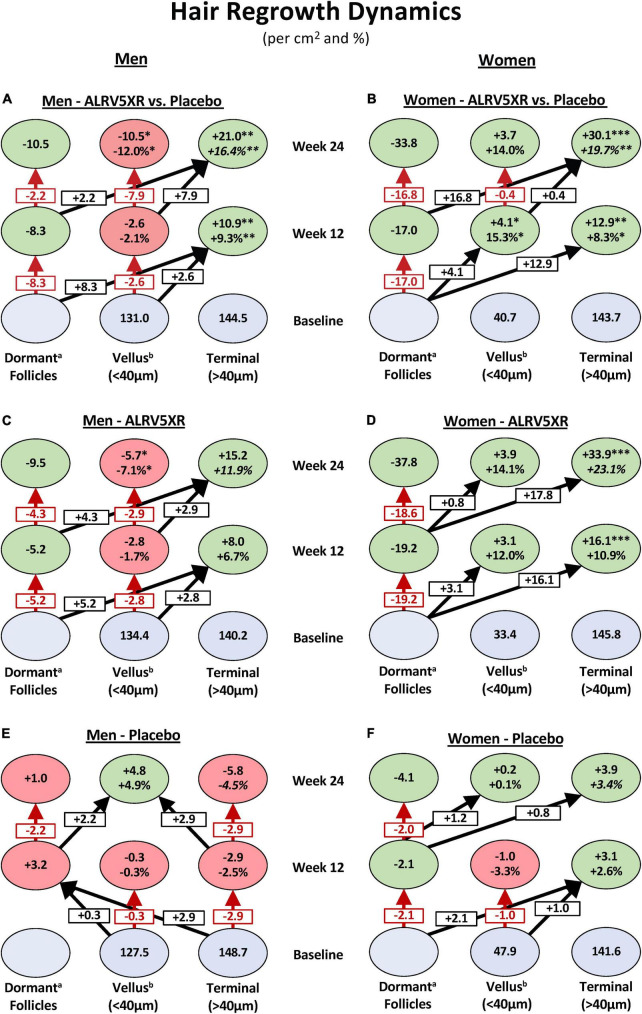
Hair regrowth dynamics of ALRV5XR vs. placebo in women and men with AGA and TE over 24 weeks. ALRV5XR vs. Placebo **(A,B)**, ALRV5XR **(C,D)**, placebo **(E,F)**. The dynamics show for women, new hair growth is primarily from dormant hair follicles, and in men, terminal hair is mostly regenerated *via* miniaturization reversal of vellus-like hair. Baseline values in blue ovals are absolute counts of vellus and terminal hair. Values at 12 and 24 weeks are absolute and relative changes in hair counts from baseline. Decreases in dormant follicles represent the total of new hairs grown. Vellus hair includes true vellus, vellus-like, and new hair. Dormant follicle changes are the sum of vellus and terminal hair changes, and any small differences in these results compared to total hair density changes are due to rounding or methods of analyzing evaluable subjects for total hair density. Dormant follicle counts at baseline are not known. Values at 12 and 24 weeks are absolute and relative changes in hair counts from baseline. For vellus and terminal hairs, green ovals represent increases in hair, and red ovals represent decreases. Decreases in dormant follicles are colored green. a: Changes in dormant follicles account for less empty follicles due to new hair regrowth. b: Vellus Hairs include True Vellus, Vellus-Like, and New Hairs. Arrows in black show new hairs from dormant follicles regrowing into vellus or terminal hairs and vellus hairs thickening into terminal hairs. Arrows in red are reductions in vellus hairs or dormant follicles. Statistical significance between ALRV5XR and placebo: *p < 0.05; **p < 0.01; ***p < 0.001.

In women, 29.8 TH/cm^2^ or 98.7% of the 30.1 TH/cm^2^ increase in hair density emerged from dormant hair follicles, and 0.4 TH/cm^2^ or 1.3% came from thickening of VH. In men, the density increase of 21.0 TH/cm^2^ was derived equally from dormant hair follicles 10.5 TH/cm^2^ (50%) and 10.5 TH/cm^2^ (50%) were derived from thickening of VH (see Figure 2).

## Discussion

Previously, for ALRV5XR, we reported remarkable and clinically significant increases in TH regrowth when compared to other treatments (1, 2). In these studies, both sexes had common TH but highly differential VH densities at baseline (see Supplementary Table S1). The observed data indicates that both the reactivation of dormant TH follicles and thickening of AGA miniaturized (vellus-like) hairs are sources of the new TH; however, there are significant differences between the sexes (see Table 1 and Figures 1, 2).

In women, the observed significant increase in TH and total hair densities and non-significant increase in VH and TH/VH ratio suggest that the women’s TH regrowth was primarily from activation of dormant TH follicles as seen by the double-digit shift in hair density toward TH at 12 and 24 weeks in Figure 2B. Similar substantial shifts (>15 Hairs/cm^2^) toward TH are seen in the regrowth dynamics within the ALRV5XR group (Figure 2D), whereas changes in the placebo group were much smaller (<2.5 Hairs/cm^2^) as seen in Figure 2F. Furthermore, the non-significant changes in diameters of total hair and TH suggest there was also some thickening of AGA miniaturized TH follicles that contributed to the diameter distribution with a thinner caliber of TH (Table 1). Therefore, in women, the dominant effect of TH regrowth was from reactivating dormant TH follicles (Figure 2).

In contrast, ALRV5XR in men saw a significant reduction in VH density along with a significant increase in TH density as seen by a net shift to TH of > + 10 Hairs/cm^2^ originating primarily from dormant HFs during the first 12 weeks and mainly from VH during the second 12 weeks as seen in Figure 2A. This same effect is seen in the ALRV5XR group, where arrows mostly point to the right in Figure 2C, but net hair shifts are <10 Hairs/cm^2^. In the placebo group, regrowth dynamics are very different, as seen in Figure 2E, with net hair loss of 3.2 Hairs/cm^2^ during the first 12 weeks and a shift toward miniaturization of TH to VH (+2.9 Hairs/cm^2^) and regrowth of VH (+2.2 Hairs/cm^2^) during the second 12-week period. Consequently, the TH/VH ratio increased significantly. In contrast, an increase in total hair density was not significant, indicating that the men’s TH regrowth was derived in equal parts from the reversal and thickening of AGA miniaturized TH follicles and regeneration of new THs from dormant HFs. Furthermore, there were no significant changes in average total and TH diameter, supporting the fact that half of the new TH came from empty TH follicles (Table 1). Therefore, in men, TH regrowth was equally from thickening of AGA miniaturized TH and regeneration of new TH from dormant HFs (Figure 2).

**FIGURE 3:**
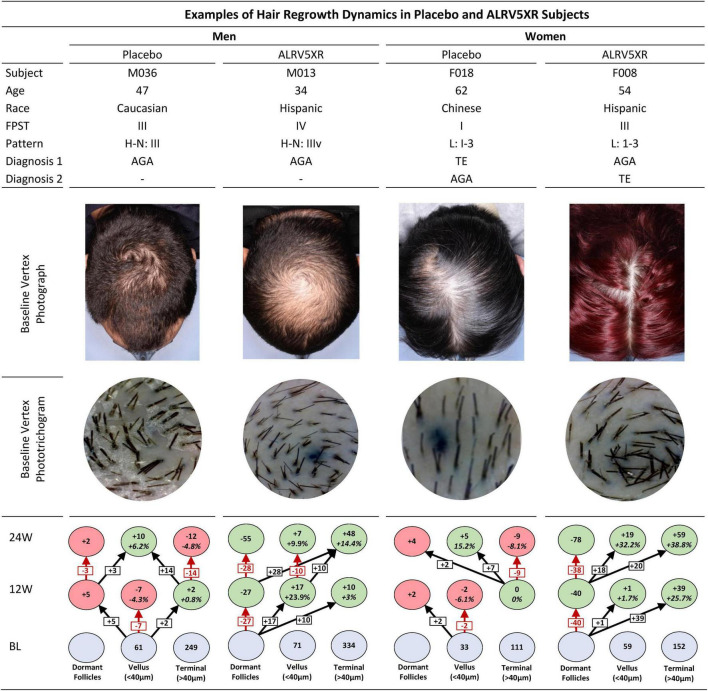
Examples of hair regrowth dynamics of ALRV5XR vs. Placebo in subjects over 24 weeks. This figure shows the individual hair regrowth dynamics of four subjects, one male and one female in placebo and ALRV5XR treatment arms. The clinical context of each subject is presented with baseline global vertex photographs and phototrichogram images. The corresponding hair regrowth dynamics chart after 12 and 24 weeks shows the placebo subjects with a progressive reduction in terminal hair density, miniaturizing of terminal hair to vellus hair, and the increasing of empty hair follicles. The ALRV5XR subjects show a completely opposite dynamic of progressive terminal hair regrowth, miniaturization reversal, and decreasing empty follicles. FPST, fitzpatrick skin type; Pattern, H-N: Hamilton Norwood; L, Ludwig; Diagnosis 1 and 2, primary and secondary diagnosis; BL, baseline, 12W and 24 are 12 and 24 weeks, respectively. Blue ovals are absolute terminal or vellus hair counts at BL. Dormant follicle counts at baseline are not known. Values at 12 and 24 weeks are absolute and relative changes in hair counts from baseline. For vellus and terminal hairs, green ovals represent increases in hair, and red ovals represent decreases. Decreases in dormant follicles are colored green. Changes in dormant follicles account for less empty follicles due to new hair regrowth. Vellus hairs include true vellus, vellus-like, and new hairs. Arrows in black show new hairs from dormant follicles regrowing into vellus or terminal hairs, vellus hairs thickening into terminal hairs, terminal hair miniaturizing into vellus. Arrows in red are reductions in vellus hairs, terminal hairs, or increases in dormant follicles.

For purposes of clinical context, four subjects, one from each trial arm were analyzed with global photographs, phototrichogram images, and individual hair regrowth dynamics charts over 24 weeks of treatment (see Figure 3).

Similar to ALRV5XR in women, the current first-line treatments for AGA (oral finasteride in men and topical minoxidil in women and men) result in TH regrowth mostly from dormant telogen TH follicles (3–6). Nevertheless, these first-line treatments are unable to reverse the miniaturization of vellus-like hairs and attain normal THs as ALRV5XR does in men (3–5). This unique property suggests a novel mechanism of action for ALRV5XR. Interestingly, during induction of hair and skin regeneration, platelet-rich plasma (PRP), autologous stem cell therapy (ACST), and ALRV5XR act as activators of similar growth factor and cytokine subcellular routes, including the Wnt/β-catenin signaling pathway (1, 2, 7). Moreover, in a related but different matter, it can be observed that senescent alopecia (SA) and AGA display similar miniaturization and histologic features (8). Consequently, the mechanism by which ALRV5XR attains reversal of those changes might illustrate distinct and specific features belonging to physiological regeneration of senescing tissues, thereby allowing the identification of potential sites for pharmacologic intervention.

The above observations suggest that despite clinically significant TH regrowth in both sexes, ALRV5XR is likely to reactivate dormant telogen follicles faster than it can reverse miniaturization of hair. Consequently, women have the potential to restore a more normal scalp hair phenotype quicker than men.

## Limitations

The inability to differentiate new hairs amongst true vellus and vellus-like hair diameters <40 μm by phototrichogram may have contributed to a more conservative TH density and an inflated VH density. Also, more extensive trials are needed to provide more statistically robust data on hair regrowth dynamics.

## Findings

ALRV5XR significantly increased TH density in both sexes without adverse effects. Regeneration of structure and function of both dormant telogen and impaired anagen follicles were documented. In women, an accelerated growth rate was observed over two 12-week treatment periods, and reactivation of dormant telogen follicles appeared to be the dominant influence leading to increased TH density. In men, while reactivation of dormant telogen follicles similarly contributed to increased TH density, a novel equally contributing ALRV5XR influence was observed. Indeed, a newfound effect, the thickening of the AGA miniaturized hair, contributed toward normalization of the physiologic hair phenotype. Prolonged treatment may restore a more normal scalp hair phenotype in women in a shorter time than in men.

## Data availability statement

The raw data supporting the conclusions of this article will be made available by the authors, without undue reservation.

## Ethics statement

The studies involving human participants were reviewed and approved by IRB-Institutional Review Board Services. The patients/participants provided their written informed consent to participate in this study.

## Author contributions

PRF and KMF contributed to the study concept and design. PRF obtained funding and drafted the manuscript. JG-A, OJF, and KMF critically revised the manuscript for important intellectual content. All authors accessed and were responsible for the data associated with the study, contributed to data validation, data analysis, statistical analysis, interpretation, and visualization, and approved the submitted version.
