# Supplementary material for: Sex differences in clinical trials of ALRV5XR treatment of androgenetic alopecia and telogen effluvium
Source: Front Med (Lausanne). 2022 Aug 4;9:918058. doi: 10.3389/fmed.2022.918058 (PMC9421616; doi:10.3389/fmed.2022.918058)
Supplement: Supplementary file 1 [file Table_1.pdf]

SUPPLEMENTARY MATERIALS

Article Title

Sex differences in clinical trials of ALRV5XR treatment of androgenetic alopecia and telogen effluvium

Peter R Feldman[1], Oriel J Feldman[1,2], Jaime Guevara-Aguirre[3,4,5,6], Klaus M Fiebig[1]

[1]Arbor Life Labs, Toronto, Ontario, Canada

[2]Faculty of Science, Wilfrid Laurier University, Waterloo, Ontario, Canada

[3]College of Medicine, Universidad San Francisco de Quito (USFQ), Quito, Ecuador

[4]Faculty of Health, Medicine and Life Sciences, Maastricht University, Maastricht, The Netherlands

[5]Institute of Endocrinology, Metabolism, and Reproduction (IEMYR), Quito, Ecuador

[6]College of Medicine, University of Florida, Gainesville, Florida, USA

*** Correspondence:**
Corresponding Author: Peter R Feldman
petefeldman@gmail.com

Accepted: 13 July 2022

Contents:

Table S1. Baseline Characteristics

Table S2. Response Rates as Change in Terminal Hairs from Baseline.

Table S3. ALRV5XR Material Active Ingredients

Table S1. Baseline Characteristics

	Men		Women	
	**Placebo** N=23(11)[a]	**ALRV5XR** N=23(11)[a]	**Placebo** N=21(21)[a]	**ALRV5XR** N=20(18)[a]
**Sex**				
Male	23 (100%)	23 (100%)	0 (0%)	0 (0%)
Female	0 (0%)	0 (0%)	21 (100%)	20 (100%)
**Age (years)**				
Mean (±SD)	45·7 (±12·9)	48·3 (±7·9)	51·6 (±10·7)	50·1 (±9·1)
Median (IQR)	48·0 (34·0 – 54·3)	47·0 (45·5 – 52·5)	48·0 (33·0 – 52·0)	47·0 (45·5 – 57·8)
Range	22 – 65	32 – 63	25 – 64	24 – 62
**Anthropometrics**				
Height (cm)[b]	172·7 (170·3 – 185·5)	175·3 (170·5 – 177·8)	158·8 (157·5 – 160·0)	160·3 (154·8 – 165·0)
Weight (kg)[b]	80·2 (69·3 – 90·8)	81·4 (73·7 – 93·6)	63·6 (58·1 – 72·3)	60·1 (55·6 – 73·9)
BMI (kg/m^2)[b]	25·1 (22·6 – 31·2)	27·1 (23·7 – 31·8)	25·3 (21·6 – 31·3)	24·4 (21·7 – 27·2)
BP (mmHg)	128·3/84·1 (±13·5/9·9)	128·1/84·4 (±21·5/17·9)	118·1/74·7 (±10·8/5·7)	118·8/76·5 (±8·3/7·0)
**Trichometry**				
Terminal Hair Density (per cm^2)	148·7 (±69·5)	140·2 (±71·4)	141·6 (±32·5)	145·8 (±32·9)
Vellus Hair Density (per cm^2)	127·5 (±57·9)	134·3 (±70·4)	47·9 (±22·6)	33·4 (±21·1)
Terminal/Vellus Hair Ratio	1·1	1·5	3·5	5·6
Diameter of All Hair (µm)	44·7 (±8·3)	40·9 (±7·0)	56·2 (±7·9)	66·3 (±7·6)
Diameter of Terminal Hair (µm)	55·1 (±7·0)	53·3 (±6·9)	65·3 (±7·3)	73·6 (±6·7)
Growth Rate (µm/24 hours)	225·2 (±45·1)	237·8 (±49·7)	312·0 (±32·1)	302·3 (±33·6)
Shed Rate (Hairs/24 hours)[c]	411·0 (±183·0)	513·7 (±286·2)	131·2 (±44·0)	125·7 (±50·0)
**Diagnosis**				
AGA Primary	23 (100%)	23 (100%)	11 (52%)	16 (80%)
AGA Secondary	0 (0%)	0 (0%)	10 (48%	4 (20%
TE Primary	0 (0%)	0 (0%)	10 (48%)	4 (20%)
TE Secondary	0 (0%)	1 (4·3%)	5 (24%)	4 (20%)
**HN / Ludwig Classification**				
I / I-1	4 (17%)	0 (0%)	3 (14%)	2 (10%)
II / I-2	2 (9%)	1 (4%)	4 (19%)	11 (55%)
IIA / I-3	2 (9%)	1 (4%)	9 (43%)	5 (25%)
III / I-4	1 (4%)	0 (0%)	1 (5%)	1 (5%)
IIIA / II-2	0 (0%)	1 (4%)	0 (0%)	1 (5%)
IIIV	5 (22%)	6 (26%)		
IV	1 (4%)	4 (17%)		
V	0 (0%)	1 (4%)		
VA	3 (13%)	2 (9%)		
VI	1 (4%)	0 (0%)		
VII	2 (9%)	3 (13%)		
Missing	2 (9%)	4 (17%)	4 (19%)	0 (0%)
**Fitzpatrick Skin Type**				
I	3 (13%)	1 (4%)	3 (14%)	4 (20%)
II	3 (13%)	2 (9%)	8 (38%)	8 (40%)
III	10 (44%)	15 (65%)	7 (33%)	8 (40%)
IV	2 (9%)	1 (4%)	2 (10%)	0 (0%)
V	2 (9%)	1 (4%)	0 (0%)	0 (0%)
VI	2 (9%)	3 (13%)	1 (5%)	0 (0%)
Unknown	1 (4%)	0 (0%)	0 (0%)	0 (0%)
**Ethnicity**				
African American	4 (17%)	4 (17%)	0 (0%)	0 (0%)
Caucasian	10 (44%)	7 (30%)	3 (14%)	1 (5%)
Chinese	5 (24%)	6 (26%)	12 (57%)	13 (65%)
Filipino	0 (0%)	1 (4%)	0 (0%)	1 (5%)
Hispanic	1 (4%)	3 (13%)	4 (19%)	3 (15%)
Indian	1 (4%)	0 (0%)	0 (0%)	0 (0%)
Japanese	0 (0%)	0 (0%)	2 (10%)	1 (5%)
Korean	1 (4%)	0 (0%)	0 (0%)	0 (0%)
Mongolian	0 (0%)	1 (4%)	0 (0%)	1 (5%)
Thai	0 (0%)	1 (4%)	0 (0%)	0 (0%)
Vietnamese	1 (4%)	0 (0%)	0 (0%)	0 (0%)

Legend: a: Evaluable for Hair Efficacy (Per Protocol); b: Median (IQR); ± or SD=standard deviation; c: Scalp Shed Rate based on 700cm^2 shedding assumed uniformly from phototrichogram area on vertex; Trichometry measures are per protocol; AGA: Androgenetic Alopecia; TE: Telogen Effluvium HN: Hamilton-Norward classification of hair loss pattern for men. Ludwig classification of hair loss pattern for women. Vellus hair includes true vellus hair, vellus-like and new hair.

Table S2. Response Rates as Change in Terminal Hairs from Baseline. This table shows the stratified response by treatment group with a substantial increase in response in hair growth in the ALRV5XR groups over time. **In Women,** the ALRV5XR group's change in terminal hair (TH) /cm^2 shows a bimodal responder pattern of non-responders and very high responders with a significant shift of responding subjects' hair count changes from a relatively even distribution in women of 0-40 TH/cm^2 at week 12 to higher values of 30 THs/cm^2 and above at week 24. Response Rates by % Change in THs for the ALRV5XR treatment group show a significant shift of subjects' hair % changes at week 12 to higher percentage ranges at week 24. **In Men**, the ALRV5XR group separates into a trimodal responder pattern of low, moderate, and high responders when measured by absolute changes in TH density; however, percent changes in TH density are more uniform. 54·5% of the ALRV5XR group increased their TH density by 10 or more THs/cm^2 by week 24 and 18·2% of the group increased their TH density by more than 25 THs/cm^2. Overall, men have a perfect response rate, while women grow significantly more hair than men.

	Men					Women				
		Placebo		ALRV5XR			Placebo		ALRV5XR	
		Week 12	Week 24	Week 12	Week 24		Week 12	Week 24	Week 12	Week 24
**Positive Responders**		50·0%	18·2%	91·9%	100%		67·7%	42·9%	77·8%	66·7%
**Terminal Hairs/cm^2**										
	<0	50·0%	81·8%	9·1%	-	<0	33·3%	57·1%	22·2%	33·3%
	0-5	40·0%	18·2%	27·3%	45·5%	0-10	47·6%	28·6%	16·7%	-
	5-10	10·0%	-	27·3%	-	10-20	9·5%	4·8%	16·7%	-
	10-15	-	-	18·2%	27·3%	20-30	9·5%	-	16·7%	-
	15-20	-	-	9·1%	9·1%	30-40	-	-	16·7%	5·6%
	20-25	-	-	-	-	40-50	-	4·8%	11·1%	22·2%
	≥25	-	-	9·1%	18·2%	≥50	-	4·8%	-	38·9%
Min Regrowth		-17	-12	-3	0		-7	-9	-9	-6
Max Regrowth		9	3	25	51		22	54	41	79
**Terminal Hairs %**										
	<0%	50·0%	81·8%	9·1%	0·0%	<0%	33·3%	57·1%	22·2%	33·3%
	0-5%	40·0%	18·2%	45·5%	45·5%	0-10%	47·6%	28·6%	22·2%	-
	5-10%	10·0%	-	27·3%	9·1%	10-20%	9·5%	4·8%	33·3%	11·1%
	10-15%	-	-	-	18·2%	20-30%	9·5%	-	16·7%	11·1%
	15-20%	-	-	-	9·1%	30-40%	-	4·8%	5·6%	27·8%
	20-25%	-	-	18·2%	9·1%	40-50%	-	4·8%	-	11·1%
	≥25%	-	-	-	9·1%	≥50%	-	-	-	5·6%
Min Regrowth		-12·1%	-12·3%	3·4%	0%		-5·3%	-8·1%	-6·0%	-4·9%
Max Regrowth		7·0%	1·6%	22·9%	46·8%		18·3%	45·0%	36·3%	69·9%

Table S3. ALRV5XR Material Active Ingredients

	Active Ingredients*
Oral (capsule)	Angelica sinensis, Ascophyllum nodosum, Astragalus membranaceus, Coenzyme Q10, Copper, Eclipta prostrata, Folate, Ganoderma lucidum, Malus domestica, Moringa oleifera, Niacin, Pantothenic acid, Polygonum multiflorum, Punica granatum, Riboflavin, Rosmarinus officinalis, Scutellaria baicalensis, Selenium, Silicon, Sophora flavescens, Vitamin A, Vitamin B6, Vitamin B7(Biotin), Vitamin B12, Vitamin D, Vitis vinifera, and Zinc
Shampoo	Caffeine, Eclipta prostrata, Lupinus, Momordica charantia, Moringa oleifera, Pyrus malus, Rosmarinus officinalis, Vitis vinifera, Scutellaria baicalensis, Vitamin B7(Biotin)
Conditioner	Caffeine, Eclipta prostrata, Lupinus, Momordica charantia, Moringa oleifera, Pyrus malus, Rosmarinus officinalis, Vitis vinifera, Scutellaria baicalensis, Vitamin B7(Biotin)
Follicle serum	Caffeine, Eclipta prostrata, Lupinus, Momordica charantia, Moringa oleifera, Ocimum basilicum, Pisum sativum, Pyrus malus, Rosmarinus officinalis, Vitis vinifera, Scutellaria baicalensis, Vitamin B7(Biotin)

*Patents pending
